# The Challenges in Clinical Diagnosis of Trigeminal Neuralgia: A Review

**DOI:** 10.7759/cureus.61898

**Published:** 2024-06-07

**Authors:** Joseph V Pergolizzi, Jo Ann K LeQuang, Salah N EL-Tallawy, Rania S Ahmed, Morgan Wagner, Giustino Varrassi

**Affiliations:** 1 Pain Medicine, NEMA Research, Inc., Naples, USA; 2 Scientific Communications, NEMA Research, Inc., Naples, USA; 3 Anesthesia and Pain Management, Faculty of Medicine, Minia University and NCI, Cairo University, Cairo, EGY; 4 Anesthesia and Pain Management, College of Medicine, King Khalid University Hospital, King Saud University, Riyadh, SAU; 5 College of Medicine, Alfaisal University, Riyadh, SAU; 6 Entrepreneur Program, NEMA Research, Inc., Naples, USA; 7 Pain Medicine, Paolo Procacci Foundation, Rome, ITA

**Keywords:** diagnose trigeminal neuralgia, migraine disorder, migraine, painful trigeminal neuropathy, post-traumatic trigeminal neuropathy (pttn), trigeminal neuralgia

## Abstract

The lack of established laboratory tests or biomarkers for trigeminal neuralgia (TN) makes diagnosing this relatively rare condition extremely challenging. Trigeminal nerve compression observable on magnetic resonance imaging may indicate TN, but many patients do not have visible lesions or compression. In particular, TN may be confused with migraine, cluster headache, temporomandibular disorder, and other types of headache. An accurate diagnosis is imperative for proper treatment since these conditions do not respond to the same treatment. Many symptoms of these headaches can be vague or overlap, and clinicians depend in large measure on the subjective reports of their patients. Nevertheless, it is imperative to diagnose TN better, which can cause excruciating pain, reduce the quality of life, and even result in disability. It is possible that TN is underestimated.

## Introduction and background

This initial challenge in trigeminal neuralgia (TN) is obtaining a prompt and accurate diagnosis to allow for effective treatment. TN is associated with severe pain, decreased quality of life, disability, and burden to the healthcare system; it remains incompletely elucidated and challenging to diagnose [1]. The stated lifetime prevalence of TN at 0.3% of the general population may be underestimated [2,3]. Most of the research and information about TN comes from North America, China, and Europe, which may provide an incomplete global picture of the epidemiology of TN.

Validated test instruments can assess pain levels associated with TN, but only artificial neural networks (ANNs) provide some guidance in orofacial pain diagnoses. ANN is an online system driven by artificial intelligence, but accurate diagnosis of TN still depends on a clinical evaluation [4]. Accurate clinical diagnoses depend on the ability of clinicians to ask the right questions and patients to report subjective impressions accurately. Since the symptoms of TN may overlap with other facial pain conditions, diagnosis can require some careful patient interviews. Furthermore, TN is sometimes comorbid with other headaches, such as migraine [5]. Diagnostic confusion can compromise care because similar symptoms in orofacial pain and headache disorders may respond to different treatments [6,7]. This is a narrative review of the clinical navigation of TN diagnosis and pitfalls.

## Review

Terminology and diagnostic categories

The main types of TN are primary, subdivided into classical and idiopathic, or secondary. Classical TN accounts for about 85% of all cases and is often associated with neurovascular compression, although this may not always be evident in imaging studies [8,9]. The most frequently observed form of secondary TN occurs in the setting of multiple sclerosis (MS) [10], which also elevates the patient’s risk for migraine headaches [11].

The diagnostic paradigms for TN describe intense, severe pain triggered by a mild stimulus, such as a gentle touch or chewing. Pain has an abrupt onset and occurs in paroxysms of seconds to minutes but repeats frequently. The pauses between paroxysms may be pain-free, or there may be a lower level of persistent underlying pain (Table 1).

**Table 1:** Diagnostic criteria for TN set forth by major specialty societies Specialty societies have defined diagnostic criteria for TN [3,7,12-14] MS: multiple sclerosis; TN: trigeminal neuralgia

Characteristic	Third edition of International Classification of Headache Disorders	International Association for the Study of Pain	European Academy of Neurology
Triggers	Innocuous stimuli along the trigeminal distribution	Innocuous stimuli along the trigeminal distribution, but pain may arise spontaneously	Not mentioned
Pain	Paroxysms with or without concomitant underlying pain	Paroxysmal pain but concomitant underlying pain is possible	May be purely paroxysmal or paroxysmal plus concomitant persistent pain
Painful paroxysms	Lasts from less than one second to two minutes. Severe intensity. Often has an “electric” or stabbing quality	Few seconds to a maximum of two minutes. Severe intensity	Painful paroxysms
Location	Along distributions of trigeminal nerve, typically unilateral	Along distributions of the trigeminal nerve, typically unilateral. TN secondary to MS is sometimes bilateral	Along distributions of trigeminal nerve, usually unilateral, but younger and secondary patients may have bilateral symptoms
Trigeminal sensory deficits	Not mentioned	Not mentioned	More common in those with secondary TN than primary

Classical TN is differentiated by evidence of neurovascular compression and/or morphological changes in the trigeminal nerve root, while idiopathic TN shows no evidence of a nerve lesion or other cause of TN [13,15]. Imaging studies of classical TN patients sometimes show evidence of neurovascular compression on the asymptomatic and affected sides [16].

Both classical TN and idiopathic TN are associated with pain paroxysms, which may or may not occur against a background of concomitant pain [15]. Secondary TN is associated with an underlying disease or condition, such as a tumor, arteriovenous malformation, or MS [13].

Epidemiology and risk factors

A genetic basis for TN has never been established but is plausible [12,17-19]. Imaging studies are routinely conducted to differentiate classical from idiopathic TN, but many such studies are unremarkable and inconclusive [16]. An observational study from Addis Ababa, Ethiopia, found that 90% of patients with diagnosed TN presented with a normal neurological examination [20]. Such results may cause TN pain to be inappropriately attributed to another cause. In a study from Abbottabad, Pakistan, of 117 TN patients, 88% previously underwent dental extraction to treat the pain. However, in many cases, this treatment did not resolve the pain [21].

The annual incidence of TN has been reported to be 4-29 per 100,000 person-years with a 3:2 prevalence in women versus men. The mean age at the onset of TN is 53-57 years, although pediatric cases have been reported [22,23]. Right-sided pain occurs in about 60% of cases, and bilateral primary TN occurs in fewer than 5% of cases [15,24]. In Western nations, the lifetime prevalence of TN is 0.16%-0.3% [25]. Often considered a rare disorder, TN may actually be underestimated [3].

The condition is slightly more common in women than men, although a small study from Ethiopia found a slightly higher prevalence in men than women [26]. It affects mostly older people; in a study of 133 classical and idiopathic TN patients in Japan, the average age of subjects was 60.4 ± 15 years, but the average age differed by sex. Among women, the average age was 63.2 ± 15 years, while men were younger, 55.3 ± 14 years [16]. However, when classical TN is analyzed separately from its idiopathic counterpart, the classical form occurs more often in women, while idiopathic TN occurs more often in men [16].

Secondary TN is not fully elucidated. The rate of TN is higher among MS patients than in the general population; about 2% of all people with TN have MS [27]. MS precedes TN in most cases, and bilateral pain is more common in this population [19,28,29].

Various small studies have found associations between TN and other conditions, such as anxiety, depression, autoimmune disorders, dementia, diabetes mellitus, and osteoporosis [30-34]. The association between TN and hypertension is equivocal. Idiopathic intracranial hypertension can elevate intracranial pressure and cause painful headache symptoms and visual perturbances, but it is not clear if this results in secondary TN [35]. In a retrospective database study of 84 patients with diagnosed TN and 252 age- and sex-matched controls, it was found that 37% of the TN patients had hypertension versus 32% of the controls, but this was not statistically significant [36].

Symptoms of TN

TN is characterized by intense paroxysms of very brief pain, which in approximately half of all cases occurs against a background of concomitant continuous but less intense pain [18,37,38]. The intense abrupt-onset pain flares of TN last only seconds to a maximum of two minutes but may recur multiple times in a short period. Ten to fifty attacks of pain paroxysms may occur in a day [17,39]. The refractory period between pain paroxysms can be brief and may be asymptomatic [3]. Background pain is more common in women and often occurs during an attack but not between attacks [3].

**Figure 1:**
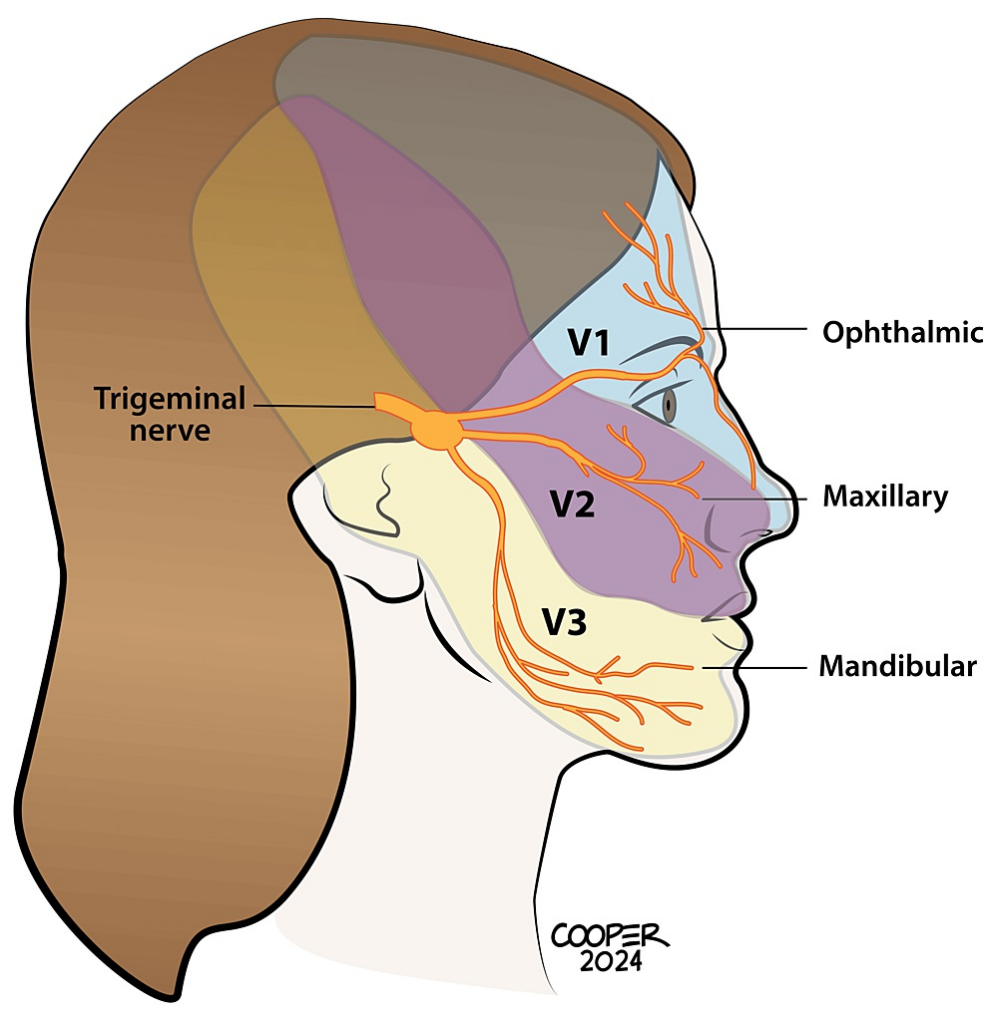
The trigeminal is the longest cranial nerve with three main distributions. Only about 5% of TN patients experience pain in the V1 region. Most patients experience painful symptoms in V2 and/or V3 This medical illustration was created by Todd Cooper of Coyote Studios, Green Valley, California. It is original and owned by the authors TN: trigeminal neuralgia

Pain can be localized along the trigeminal nerve distribution regions, with the rarest TN attacks occurring in the V1 region. Pain may occur in more than one region (V1+V2 and V2+V3), with the latter being the most common (Figure 1) [17,40,41].

Sensory aberrations have been documented in about 15%-25% of TN sufferers [42] and include higher thresholds for touch and temperature in the affected nerve region, while adjacent regions exhibit only tactile alterations [43,44]. Bilateral hyperalgesia for temperature and touch has been reported even in patients with unilateral TN [45].

Clinical diagnosis

The clinical diagnosis of TN is predicated on a thorough patient history and interview. There are no established diagnostic or laboratory tests. Primary TN patients may present with normal physical and neurologic results, although minor sensory abnormalities may exist [17].

After a thorough patient history, patients should be asked to describe their painful symptoms, including the localization of pain, its duration and frequency, and a description of pain quality. TN responds to mild triggers, which the patient may be able to report in detail. Patients may lack the vocabulary to describe subjective symptoms and may be unfamiliar with how to describe various types of pain. Without leading the patient, the clinician may want to ask highly detailed and specific questions and provide some minor prompts (Table 2).

**Table 2:** Suggested questions for a patient interview about the characteristics of pain during attacks TN: trigeminal neuralgia

Question	Responses suggesting TN
Where do you feel the pain?	Pain may be in any part of the trigeminal distribution but is likely in V2 and/or V3
Do you have pain on one or both sides?	Pain is typically unilateral and consistent on that side
Does anything like a touch or chewing food trigger pain?	Yes
Do you feel any pain inside your mouth?	This suggests a dental problem that must be ruled out; it may also occur in TN
How long does the pain last?	Trigeminal pain has abrupt onset and short paroxysms, from seconds to two minutes
Between bursts of pain, do you have any underlying pain?	Ambient pain may occur in TN between paroxysms in an attack but does not occur after the attack is over
How many bursts of pain do you have? (Patient may be encouraged to estimate)	10 to even 50 bursts may occur
On a scale from 0 to 10, how severe would you say the pain is?	Pain is typically moderate to severe and is often very severe
How would you describe the pain? (If needed, prompt words might include the following: dull, sharp, “electric,” stabbing, deep, throbbing, mild, “pins and needles,” and so on)	Pain is often stabbing, sharp, or maybe electric
When you experience this attack, do your eyes water or does your nose run?	TN is not commonly associated with autonomic symptoms, and this suggests other types of headaches that must be ruled out.
Does anything lessen the pain?	TN does not usually respond to triggers, sleep, darkness, or other attempts to soothe the pain
Do you pace or feel restless during an attack?	This is more common for cluster headaches, which should be ruled out; it may occur in TN but is not common
Can you sense an attack coming on or does it come on suddenly?	Migraines are often sensed in advance, while TN occurs suddenly
Does the pain start out severe or does it build up slowly?	TN pain typically starts out at maximum intensity, while migraine and tension-type headaches can build slowly

Patients should be asked about their personal history of these attacks, and many patients will have distinct recollections of when the pain first occurred [46]. TN is associated with evoked pain caused by a mild mechanical stimulus on or near the face or gums, such as touch, chewing, brushing the teeth, eating, drinking, or washing the face [17,47]. While spontaneous pain may occur within an attack of TN, evoked pain is a diagnostic requirement [12]. Note that in patients with comorbid conditions, such as migraine plus TN, the painful symptoms may blur together in the patient’s mind as one disorder, so patients may report that “sometimes” a touch can trigger an attack, but other times, the attack comes on slowly.

When pain is paroxysmal and unilateral and can be triggered by a gentle touch or mild stimulus, TN should be suspected [48]. While neurovascular compression can cause TN, some patients with TN show no evidence of neurovascular compression, and some people with neurovascular compression exhibit no signs or symptoms of TN [16]. While lacrimation may occur with TN, its rarity means it has no particular utility in diagnosing [12]. Autonomic symptoms are more common in clusters and other types of headaches [3].

A careful patient history is necessary because it is important to ascertain if the pain was preceded by some kind of trauma, oral surgery, or infection, particularly herpes zoster. Comorbid conditions, in particular MS, should also be considered. A general clinical and neurologic examination is recommended but may be unremarkable [3]. The patient should then undergo laboratory testing (routine blood panels for electrolytes, liver, and kidney function), an electrocardiogram, and a magnetic resonance imaging (MRI) of the brain and brainstem [3].

In rare instances, facial pain may be associated with giant cell arteritis or cancer that triggers a neuropathic pain condition. Burning mouth syndrome is a form of neuropathic pain and occurs most frequently in perimenopausal women but may be confused with facial pain syndromes [48]. In making a differential diagnosis, it is important to consider the main types of conditions (Table 3).

**Table 3:** Points to consider in differential diagnosis of TN Differential diagnosis of TN [49] MRI: magnetic resonance imaging; TMD: temporomandibular disorder; TN: trigeminal neuralgia; SUNA: short-lasting unilateral neuralgiform; SUNCT: short-lasting unilateral neuralgiform headache with conjunctival injection and tearing

Description	TN	Dental	TMD	Cluster	Migraine	SUNA/SUNCT	Paroxysmal hemicrania
Pain timing	Paroxysmal, short duration, and repeated	May be associated with recent dental work; pain can last a few months	Hours or continuous, may be episodic	Paroxysmal episodes of short duration that cluster for an attack that can persist for hours	Gradual onset may be preceded by aura; pain is continuous throughout the attack that can last hours	Paroxysms of short duration but severe pain that repeats; no continuous background pain	Episodic bursts of pain lasting 2-30 minutes may repeat multiple times a day
Remissions	Yes, maybe pain-free between paroxysms and often fully asymptomatic between attacks	Yes, will be persistent but resolve	Can persist for years	Yes, unexpected remissions occur	Yes, possible	Yes, unexpected remissions occur	Yes, unexpected remissions occur
Pain quality	“Electric,” shooting, stabbing pain in the face. Pain starts suddenly and at severe intensity. Background pain may or may not be present between paroxysms	Sharp pain, deep aching pain, and throbbing sensation	Mostly deep, dull pain, but may be sharp at times. Builds up gradually. Can be mild or severe, or can be chronic	Sharp stabbing pain around one eye, sudden onset, and severe intensity. Pain may remit entirely between episodes	Gradually building pain that can become severe in intensity; no paroxysms or flares of pain. Persistent over several hours	Sharp, stabbing, burning pain of severe intensity and short duration (five seconds to a few minutes) with remissions and repetitions	Deep pain, throbbing sensations, sometimes stabbing, or shooting pain. Pain is episodic (2-30 minutes) and can repeat many times over the course of a day
Pain location	Along trigeminal distribution (V2 and/or V3 mostly), but intraoral sometimes occurs. Frequently unilateral	Intraoral and localized around a tooth	Around the mandible but may radiate up (temple) or down (neck)	Typically unilateral, around one eye	May be unilateral or bilateral	Periorbital but may extend to the maxillary region	Periorbital that can extend to temple
Triggers	Light touch and innocuous triggers. Evoked pain is necessary for diagnosis	Hot, cold, and sweet applied to the tooth; eating may cause pain as well	Chewing, grinding teeth, yawning, and eating	Food triggers (red wine, chocolate), bright light, loud sounds, but triggers not necessary	Food triggers (red wine, chocolate), bright light, loud sounds, but triggers not necessary	Light touch	None known
Often confused with	Migraine or cluster	TMD	Dental	Paroxysmal hemicranias and TN	TN	TN, migraine	Migraine
Differential considerations	MRI studies may be useful. Abrupt onset	Patient history and dental referral	Pain location and oral involvement	Restlessness and agitation; autonomic symptoms	May be preceded by aura; nausea often occurs with an attack	Restlessness and agitation; marked autonomic symptoms	Autonomic symptoms. Indomethacin relieves it

Understanding the various headache disorders that may present with symptoms suggestive of TN may be helpful in making a differential diagnosis (Table 4).

**Table 4:** Specific attributes related to TN compared to other conditions and severe headaches Attributes of face pain and headaches [3,15,50-65] CPH: chronic paroxysmal hemicrania; PHN: postherpetic neuralgia; TMD: temporomandibular disorder; TN: trigeminal neuralgia; SUNA: short-lasting unilateral neuralgiform; SUNCT: short-lasting unilateral neuralgiform headache with conjunctival injection and tearing

Attribute	Feature	Likely condition(s)
Autonomic symptoms	Prominent	Cluster headache, SUNCT, CPH
	Variable	Migraine, TN, PHN (mild if at all)
	None	Giant cell arteritis, dental pain
Duration of attack	Continuous pain over prolonged period	PHN, giant cell arteritis, migraine, sometimes dental pain
	Minutes only	SUNA/SUNCT, cracked tooth, TN
	Minutes to 45 minutes	CPH
	Repeated brief attacks	Cluster headache, TN
	Persistent, unrelenting	TMD
Pain location	Unilateral	TN, migraine, TMD
	Forehead	SUNA/SUNCT, CPH, PHN, giant cell arteritis
	Retrobulbar	TN (unilateral), cluster headache (unilateral) SUNCT, CPH, PHN, giant cell arteritis
	Cheek, chin	TN, migraine, dental, TMD
	Temple	Tension-type headache, giant cell arteritis, TMD
	Intraoral	Dental, TN
	Ear	TMD
Paroxysmal pain	Only paroxysms	TN, SUNA/SUNCT
	Paroxysms against background of continuous pain	Cluster headache, sometimes TN, sometimes dental pain, PHN
Triggers	Yes	Cluster headache, migraine, TN, dental pain, possibly PHN
	No	Giant cell arteritis
Other factors	Sex	Migraine is more common in women, SUNCT is almost exclusively in women, cluster headache is more common among men
	History	Dental procedures or trauma can precede dental pain; PHN requires a history of shingles
	Restlessness, agitation	Cluster headache

Neuroimaging and electrophysiologic testing

MRI studies are often recommended along the diagnostic pathway for a person with TN, although results from these images may be unremarkable and not helpful in diagnosing. The European Academy of Neurology (EAN) recommends MRI using three high-resolution sequences as part of the examination for diagnosing TN. If MRI is not possible or contraindicated, trigeminal reflexes can be substituted [7]. Cranial nerve function should be assessed, as tumors and MS are often predisposing conditions. The length of the trigeminal nerve can make imaging difficult. In most cases, the entire length of the nerve should be imaged for optimal results unless the clinician only seeks to establish whether or not there is damage only to the root entry region [56].

A cerebral MRI can determine if there is vascular compression on the trigeminal nerve. The angle formed between the trigeminal nerve and the pons or the angle between the trigeminal region and the pontine can be an important landmark. If this angle is particularly sharp or acute on the affected side and differs from the angle on the unaffected side, it may indicate TN. In an imaging study of 30 TN patients, 24 had such a sharp angle only on the affected side [57].

High-resolution MRI has demonstrated that, in some cases, neurovascular compression at the trigeminal nerve root is associated with symptoms of TN. Anatomical changes in the nerve, such as demyelination, flattening, atrophy, or other, have also been observed [58]. Indeed, myelin damage and concomitant neuroinflammation are associated with neurovascular compression [59]. Neuroimaging can help rule out dental problems, occipital neuralgia, cerebral aneurysms, tumors, and intracranial hemorrhage [6].

 A recent study found that functional MRI (fMRI) studies while the patient is at rest can show certain functional brain changes, but the diagnostic power of such fMRIs remains controversial. In a study comparing 85 people with classical TN against 79 age- and sex-matched controls, more brain entropy was observed in the thalamus and brainstem in classical TN patients than controls, while these patients exhibited less brain entropy in the inferior portion of the semilunar lobule. When a machine learning tool was utilized, it could accurately and effectively differentiate patients with classical TN from controls using brain entropy findings [60]. Gray matter volume in specific brain areas was also less in patients with classical TN compared to healthy controls, suggesting classical TN results in structural and functional brain changes and may rightly be considered a brain disorder [60].

A structured literature review reported that imaging studies can detect neural differences between the affected and unaffected sides in TN patients using structural characteristics and diffusion measurements. This suggests that people with TN experience some form of trigeminal nerve damage [61]. While MRI is the recommended imaging technique for TN, computed tomography (CT) cisternography scans may be used in patients for whom an MRI is impossible [62]. MRI tends to exhibit a higher diagnostic yield in cases of trigeminal neuropathy, but CT better displays the anatomy of the base of the skull, which may be helpful in cases of nerve injury [56].

Electrophysiologic testing can be used to differentiate temporomandibular disorder (TMD) symptoms from other types of facial pain syndromes [63].

Differential diagnosis by symptomology

Accurately describing symptoms is necessary to help establish a reasonable diagnosis, although diagnoses can be complicated by poor patient reporting or patients with comorbid conditions. Atypical face pain, chronic paroxysmal hemicrania, cluster and migraine headaches, and postherpetic neuralgia may present with symptoms similar to those of TN. Dental pain and temporomandibular pain are associated with jaw pain and possibly a history of oral surgery or dental problems; likewise, postherpetic neuralgia requires a preceding case of shingles [16,50-55]. Note that a particular challenge in clinical diagnoses is that patients experience these symptoms subjectively and may not always be able to describe them accurately.

Trigeminal autonomic cephalgia (TAC) is a broad category of several specific headache types, any of which can sometimes mimic TN. The five TAC headaches are cluster, paroxysmal hemicrania, short-lasting unilateral neuralgiform (SUNA), short-lasting unilateral neuralgiform headache with conjunctival injection and tearing (SUNCT), and hemicrania continua. While it can be clinically serviceable to group TAC disorders in this way, these are distinct headache forms with important distinctions and different first-line treatments [66]. All TACs are characterized by primarily unilateral pain, which can be very severe [67].

Diagnostic pearls and pitfalls

Odontogenic pain must be ruled out because it can produce symptoms that mimic TN. Patients suspected of having dental pain, either as an exclusive or concomitant diagnosis, should be referred to a dental specialist [3]. The patient will often remember having oral surgery, a prior toothache, or trauma. Likewise, pain that seems to originate at or near the teeth may indicate a dental problem, even if the patient is unaware of an event that might have caused dental pain.

A herpes zoster rash in the trigeminal region can cause secondary TN; patients should be asked if their initial onset of symptoms occurred at or right after such a rash [3]. Note that postherpetic neuralgia requires a recent prior case of shingles, which, in turn, requires that the patient have varicella years earlier.

TN may arise as a post-traumatic condition, such as following oral surgery or an accident. In such cases, sensory abnormalities and loss or gain of function usually correspond to a damaged peripheral nerve [68]. Post-traumatic TN is characterized by short, intense bursts of pain induced by a trigger point [3].

Bilateral pain is very rare in primary TN. Constant bilateral pain is possible in TN but more likely suggests temporomandibular joint disorder, idiopathic facial pain, or tension-type headache [3]. On the other hand, patients with MS have a somewhat more frequent incidence of bilateral TN, but it is relatively rare, even in secondary TN.

Stabbing-like pain in the scalp or around the eye may indicate occipital neuralgia, primary stabbing headache, or paroxysmal hemicrania [3]. So-called “ice pick headaches” are an emerging category of primary and secondary headache disorders and are characterized by paroxysms of sudden stabbing pain in the head with no other symptoms. Paroxysms last only a few seconds but repeat over the course of the day. Pain in ice pick headaches typically migrates from left to right or front to back (or reverse) over the course of an episode [69]. Primary stabbing headache has been recently added to the third edition of the International Classification of Headache Disorders [70]. These are ultrashort headaches with intense pain, but the pain's brief duration and migrating nature allow for ready differentiation from TN.

Thunderclap headache is a sudden-onset headache with immediate pain at maximum severity and demands urgent emergency care as it may signal subarachnoid hemorrhage, cerebral vasoconstriction, or other potentially life-threatening conditions. Diagnosis requires brain imaging and lumbar puncture if the brain image is equivocal [71].

Unilateral attacks that change sides are less indicative of TN and more likely to be cluster headaches, which typically involve pain around the orbital or supraorbital region rather than the trigeminal nerve distribution [72]. Although relatively rare, cluster headaches are the most common form of TAC [65].

The natural history of TN

There is a paucity of literature on the natural history of TN, likely because the condition is relatively rare and is not always appropriately diagnosed. A study of 25,174 TN patients found that the incidence of tinnitus was 18.21 per 100 person-years compared to 13.17 per 100 person-years for those without TN. The hazard ratio was 1.68 relative to the control group [73].

A curious characteristic of TN is periods of spontaneous remission in which the patient unexpectedly becomes pain-free [15,28]. About 20% of patients never experience remission at all, and only 6% have prolonged remissions of a year or more [74]. It is unknown why remissions like this occur or how one might encourage remission.

Although evidence is not robust, TN may not be progressive, and symptoms do not worsen over time [75-77].

Differentiation: trigeminal neuropathy

Trigeminal neuropathy produces chronic pain that is typically described as dull, deep, or aching in contrast to the sharp paroxysms of TN. Some trigeminal neuropathy patients may report throbbing pain, which tends to be persistent or longer lasting than the short bursts of pain of TN. Diagnosis may be complicated because TN may occur concomitantly with trigeminal neuropathy. Typically, the pain of trigeminal neuropathy is milder than that of TN [15].

Differentiation: TACs

Several primary headache symptoms grouped as TAC are characterized by unilateral pain with ipsilateral cranial autonomic features. TAC includes cluster headaches, paroxysmal hemicrania, SUNCT, SUNA, and hemicrania continua. TACs should be differentiated from TN, where pain follows the trigeminal nerve distribution, although the vagus nerve may sometimes be involved in TN as well [66]. The confusion between TACs and TN occurs because both are unilateral, episodic, and characterized by very severe pain. TACs, by definition, have autonomic features, such as rhinitis and lacrimation, but such symptoms in TN are far less pronounced if they even occur at all [48].

TACs are relatively rare, and little is known about them in the literature for diagnostic and clinical guidance [78]. The most common form of TAC is chronic cluster headache, which is a rare form of headache [78].

Differentiation: secondary TN

The EAN guideline reports that no particular clinical features have a high sensitivity in terms of differentiating secondary TN from primary classical or idiopathic TN. Classical TN is the most common form, and secondary TN disorders are associated with an underlying disease (such as MS), trauma, or other factors. Secondary TN is more likely to occur in younger patients, those with bilateral symptoms, or those with sensory deficits along the trigeminal nerve region [7].

Secondary TN is most frequent and has been best studied in the context of MS; this form of secondary TN is diagnosed with an MRI to determine the presence of pontine demyelinating plaques. In some cases, neurovascular compression is also found but is not a differentiating factor [79]. In the setting of MS, secondary TN is more frequent in women and occurs more often on the right side. The age at onset of TN secondary to MS is in the range of 40-50 years, that is, younger than primary TN [10,80]. More patients with TN secondary to MS have bilateral pain (18%) compared to those without MS [10,80,81]. Trigeminal sensory deficits may be observed in this population, but it should not be the diagnostic criterion because MS patients with secondary TN sometimes have no sensory limitations [82,83]. MS suggests demyelinating lesions rather than neurovascular compression, but some studies challenge that and report that neurovascular compression may occur concurrently with focal demyelination [84-87].

Note that people with MS are also at an elevated risk for migraine, which must be differentiated from TN in the secondary diagnosis [88]. Migraines are threefold more frequent among MS patients than controls [88]. In addition, people with MS are at higher risk for both cluster headaches and tension-type headaches [89].

Comorbid migraine and TN

Clinical diagnosis of TN comorbid with migraine can be challenging because the patient, on whose subjective account a diagnosis depends, may view these two distinct and medically unrelated conditions as one disorder. Such patients may report variable and contradictory symptoms, such as different pain locations or intensities. A population-based cohort study from Taiwan’s National Health Insurance Research Database data found that patients with migraines with aura were at greater risk of developing TN than those without aura. Among migraineurs overall, the hazard ratio for TN was 6.72 (p <0.001), indicating a surprisingly high risk [5].

Few high-quality studies explore the potential association between migraine and TN. Results from one database study suggest that 9% of patients with migraine also have TN [17,37]. This may be explained by the fact that peripheral trigeminovascular activation plays a role in migraine [90].

Among migraine patients with comorbid TN, the mean age at diagnosis of TN was 53.6 years compared to 50.0 years in the control group [5]. In propensity matching analysis, the risk of TN among migraineurs increased as much as sixfold in some cases over a three-year follow-up. Age, sex, diabetes mellitus, and hypertension did not affect the association of migraine with TN [5].

Is there a headache spectrum?

TN attacks are provoked by a trigger, which can be very mild or innocuous, such as a light touch, normal talking, and speaking. Triggers may be a helpful way to differentiate TN from many other types of headaches, but it is a characteristic shared by the SUNCT/SUNA headaches. While SUNCT/SUNA headaches have innocuous triggers, they tend to have very prominent autonomic symptoms, which are less pronounced than TN if they occur at all [15]. Since treatment with sodium-channel blockers can be effective for both TN and SUNCT/SUNA headaches, similar mechanisms may be involved [91]. This has given rise to the hypothesis that SUNCT, SUNA, and TN may be the same condition but on different points of one spectrum [91].

Pain is severe for all three types of headache (SUNCT, SUNA, TN headache), but locations vary. TN is more likely to occur in V2 and/or V3 than in V1, while SUNCT and SUNA are more likely to occur in V1. Autonomic features are the characteristics of SUNCT and SUNA but are rare in TN. Pain attacks have an abrupt onset and last from seconds to a few minutes, but while TN has periodic attacks followed by a refractory period, the pain in SUNCT and SUNA tends to be more continuous (Table 5) [91]. Note that because the trigeminal nerve is a common pathway for both SUNCT/SUNA and TN, it may blur the distinction between what may be distinct disorders [92]. On the other hand, SUNCT, SUNA, and the headaches of TN may share important mechanistic similarities that may aid in better treatment.

**Table 5:** Differential features between SUNCT/SUNA headaches and TN SUNCT/SUNA headaches and TN may be different aspects of the same type of disorder [64], and such a nosology would merge cephalgia with neuralgia [92] SUNCT: short-lasting unilateral neuralgiform headache with conjunctival injection and tearing; SUNA: short-lasting unilateral neuralgiform; TN: trigeminal neuralgia

Feature	SUNCT/SUNA	TN
Autonomic features	Yes, pronounced	Mild, if they occur at all
Location	More likely V1; unilateral	More likely V2 and/or V3; unilateral
Moving neck	May cause an attack	Will not cause an attack
Onset of pain	Abrupt	Abrupt
Pain paroxysms	May occur, but pain is more persistent	Brief (seconds to minutes) with refractory periods or less intense ambient pain between attacks
Time of attack	Daylight more frequent than night	Not specific
Trigger	No	Yes, tends to be a mild stimulus to the trigeminal nerve dermatome

Discussion

The trigeminovascular system remains a relatively unexplored yet vital physiologic region in medical science. Intriguing comorbidity associations suggest the importance of this region (see Table 6 in the Appendix). Positioned at the point where the nervous system intersects with the vascular system, the trigeminovascular system is highly sensitive to blood flow and subtle sensory inputs. The trigeminovascular system is involved in several painful and even debilitating disorders: migraine, TN, TAC headaches, and subarachnoid hemorrhage [93]. But beyond painful conditions, this system may regulate blood flow to the brain, acting as a vasodilator [93]. The so-called “vascular headaches,” including cluster headaches and migraine, originate not in the face or periphery but rather in the brain. Vascular headaches result when cranial circulation drives vascular dilatation and provokes pain around the trigeminal nerve. It has been proposed that the many related types of headaches evaluated in differential diagnosis should be considered neurovascular headaches [94]. In fact, certain specific headache disorders may be related to each other in ways that position them as points along a spectrum rather than as discreet disorders. This remains a speculative nosology but one that may aid in finding appropriate treatments.

The core concept of such trigeminovascular-type headaches involves the interface of neural innervation with cranial circulation, that is, how the nervous system interacts with the vascular system [95]. Despite the importance of intracranial nociception to headaches, there is a paucity of studies on the subject. This neural innervation and cranial circulation interface would explain the many similarities and diagnostic challenges encountered in primary and secondary headaches, TN, migraine, and other conditions. Clinically, these headaches are distinct and have different effective first-line treatments, yet they all rely on the same structural foundational mechanisms [96].

It had long been assumed that the brain was largely insensitive to pain signals and only certain of the largest cerebral vascular structures had any nociception. This has been challenged by recent studies that suggest that, in some cases, small blood vessels in the brain and pia mater may respond to painful stimuli [96]. The trigeminal nerve distributions innervate the supratentorial dura mater [96]. The three large dermatomes of the trigeminal nerve, known as V1, V2, and V3, are connected by way of the dura mater to a distinct intracranial region. It has long been established that intracranial pain perception is exclusively nociceptive [96,97]. MRI scans show that the V1 neurons have a superomedial location, V2 neurons are below, and V3 neurons are posterolateral [98]. Trigeminal neuroinflammation plays a crucial role and may trigger various types of headache pain [99].

As TN and other forms of primary and secondary headaches become more thoroughly elucidated, the purely clinical diagnosis of TN may give way to more reliable diagnostic methodologies or even biomarkers [100]. At present, diagnosis is made primarily using a clinical method supplemented with imaging, resulting in much confusion, particularly as subjectively reported symptoms may overlap. Since treatments vary by type of headache, reliable diagnostic techniques are urgently needed to allow for rapid and optimal treatments. See Table 7 in the Appendix for a short summary of diagnostic differentials.

The burden of headaches and facial pain on the global healthcare system is great and on the increase. Headaches are associated with lost productivity, restrictions in physical and social activities, decreased quality of life, and the fact that they can interfere with sleep and certain mental health conditions [101]. The problem should not be trivialized, as approximately 46% of the world population has an active headache disorder [102]. Greater research, including accurate diagnosis of various headache types, treatment, and structured headache clinical services, is needed in countries worldwide [103].

## Conclusions

TN is an intensely painful condition that can be challenging to diagnose. While imaging techniques may reveal the neurovascular compression indicative of primary TN, many MRI and other scans are unremarkable in patients with TN. Effective treatment depends on accurate diagnosis, which is largely made on a clinical basis. Migraine and cluster headache are often confused with TN, although in some patients these various types of headache can be comorbid. Differential diagnosis is important and further study is needed as headaches of all types are highly prevalent around the world and represent a major burden to the healthcare system.

## Appendices

**Table 6:** Smaller studies have established associations between trigeminal neuralgia and other conditions Conditions appear in alphabetical order [30-34]

Condition	Study	Conclusions
Anxiety and depression	Mendelian randomization database analysis	These conditions significantly increased the risk of trigeminal neuralgia. However, years of education emerged as a protective factor against trigeminal neuralgia in this population
Autoimmune disorders		
Dementia	Case-matched study	Dementia incidence was 4.47-fold greater in trigeminal neuralgia patients than in controls and was a greater risk in younger trigeminal neuralgia patients than older ones
Diabetes mellitus	Case-matched study	21.9% of trigeminal neuralgia had diabetes versus 12.9% of controls (p = 0.01)
Osteoporosis	Case-matched study, 14 years	Osteoporosis patients had 0.60 cases of trigeminal neuralgia per 1000 person-years compared to 0.18 per 1000 for controls

**Table 7:** Specific differentiating factors for trigeminal neuralgia Trigeminal neuralgia differential diagnosis [16,50-55]

Condition	Defining symptoms of this condition	How this condition differs from trigeminal neuralgia
Atypical facial pain	Atypical face pain has no established diagnostic criteria. Pain is intense, deep, and persistent and may be difficult for the patient to localize. May be unilateral or bilateral. Considered to be rare	Unrelenting pain. Does not generally follow trigeminal nerve distribution. May cause paresthesia, allodynia, tenderness, warmth, and numbness. No facial trigger points
Chronic paroxysmal hemicrania	Chronic, unilateral headache with ipsilateral, cranial autonomic symptoms. Restlessness and agitation may occur. Present in the forehead or retrobulbar, lasting 2-45 minutes with prominent autonomic symptoms. Indomethacin is effective against pain	Chronic pain, no abrupt-onset paroxysms. Does not generally follow trigeminal nerve distribution. Autonomic symptoms are prominent but may or may not occur in trigeminal neuralgia. Imaging studies may aid in diagnosis. Trigeminal neuralgia does not respond to indomethacin
Cluster headache	Unilateral pain around the eye with sudden onset and paroxysms of potentially severe pain with restlessness. Sleep may exacerbate the condition. Rare condition, more prevalent in men than women	While shooting pain occurs, there is a background of ambient pain; attacks last 20 minutes to hours. Autonomic symptoms and restlessness are hallmarks of cluster headache. Cluster headaches may show diurnal and seasonal rhythms. Cluster headaches may be relieved with high-flow oxygen gas, which will not benefit the trigeminal neuralgia patient. Suicidal ideation may occur with cluster headache attacks. Sleep exacerbates cluster headache; patients may wake up with a headache
Dental pain	Dental pain does not occur in paroxysms but is more consistent; it is often dull and deep and is localized intraorally. Dental pain may be triggered by eating, chewing, or temperature. In some patients, dental extraction or other oral surgery precedes the development of true trigeminal neuralgia. Note that dental pain may occur independently of trigeminal neuralgia and complicate symptomatology. Autonomic symptoms do not occur with dental pain	Pain localization may be helpful; dental pain occurs in the mouth around the tooth, while trigeminal neuralgia is in the face along the trigeminal dermatomes. Dental pain is usually preceded by a memorable event such as oral surgery, trauma, or other incident. Referral to a dentist may be appropriate
Giant cell arteritis	Pain typically is localized in the forehead, neck, or temple. Pain is continuous, and there are no paroxysms or triggers. Pain is deep, dull, and continuous	Pain does not follow the expected regions of the trigeminal nerve. No paroxysms and no obvious triggers. Pain is not sharp or stabbing
Migraine	Unilateral, often intense pain. The patient may sense an oncoming attack; some migraineurs have “aura” preceding the headache. Triggers may include food (chocolate, red wine, and certain cheeses), bright lights, and loud noise. May align with the menstrual cycle. A complicating factor is that migraines may be comorbid with trigeminal neuralgia such that patients experience these two types of headaches as one disorder	Trigeminal neuralgia is never associated with aura and has an abrupt onset. Migraine can occur with or without a trigger but does not occur with triggers such as chewing or light touch. Migraines can occur in pediatric patients and are common in younger patients than trigeminal neuralgia. Only migraines synch with menstrual cycles
Neuropathy of sensory fibers	Numbness in the face may occur along the trigeminal distribution. Pain, if it occurs, is continuous and may be mild to severe. No paroxysmal pain	No sudden-onset severe attacks of very brief pain, repeating over a period. Pain does not flare in multiple paroxysms. No triggers
Postherpetic neuralgia	Present mostly in the forehead, around the eye, rarely along the cheek, and is characterized by continuous pain, although paroxysms of pain may also occur. Unilateral or bilateral. Pain is often mild to moderate and can vary over time. There may be sensory impairment around the affected area	Pain likely does not follow trigeminal nerve distribution. Pain can be mild to severe, and variations can occur. Patient will have a history of shingles
SUNA, SUNCT	Localized in or around the forehead, retrobulbar, with shooting paroxysms, five or many minutes. Prominent autonomic symptoms. Rare disorder and almost exclusively in women	Trigeminal neuralgia does not have prominent autonomic symptoms. May present in the forehead
Temporomandibular disorders (TMD)	Myalgia and headache can occur in the context of temporomandibular disorders; pain typically is localized in the jaw, temple, and ear. Pain is modified by jaw movements and can increase with the maximum unassisted opening of the mouth. Jaw motion may be limited. Pain is persistent and may be mild to severe. Pain may be present in the form of headache	One of the most common forms of orofacial pain. Pain with temporomandibular disorders tends to be persistent, not triggered or paroxysmal. May be unilateral and overlap with trigeminal nerve distributions. Chronic TMD pain may be associated with fatigue, depression, and poor sleep

## References

[1] Gambeta E, Chichorro JG, Zamponi GW (2020). Trigeminal neuralgia: an overview from pathophysiology to pharmacological treatments. Mol Pain.

[2] Mueller D, Obermann M, Yoon MS (2011). Prevalence of trigeminal neuralgia and persistent idiopathic facial pain: a population-based study. Cephalalgia.

[3] Maarbjerg S, Di Stefano G, Bendtsen L, Cruccu G (2017). Trigeminal neuralgia - diagnosis and treatment. Cephalalgia.

[4] Teshima T, Zakrzewska JM, Potter R (2023). A systematic review of screening diagnostic tools for trigeminal neuralgia. Br J Pain.

[5] Lin KH, Chen YT, Fuh JL, Wang SJ (2016). Increased risk of trigeminal neuralgia in patients with migraine: a nationwide population-based study. Cephalalgia.

[6] Zeng C, Zhang C, Li YH, Feng X, Zhang MJ, Xiao RH, Yang HF (2021). Recent advances of magnetic resonance neuroimaging in trigeminal neuralgia. Curr Pain Headache Rep.

[7] Bendtsen L, Zakrzewska JM, Abbott J (2019). European Academy of Neurology guideline on trigeminal neuralgia. Eur J Neurol.

[8] Ge X, Wang L, Pan L, Ye H, Zhu X, Feng Q, Ding Z (2022). Risk factors for unilateral trigeminal neuralgia based on machine learning. Front Neurol.

[9] Cruccu G, Di Stefano G, Truini A (2020). Trigeminal Neuralgia. N Engl J Med.

[10] Cruccu G, Biasiotta A, Di Rezze S (2009). Trigeminal neuralgia and pain related to multiple sclerosis. Pain.

[11] Mohammadi M, Kankam SB, Salehi S (2023). The association between multiple sclerosis and migraine: a meta-analysis. Mult Scler Relat Disord.

[12] Bendtsen L, Zakrzewska JM, Heinskou TB (2020). Advances in diagnosis, classification, pathophysiology, and management of trigeminal neuralgia. Lancet Neurol.

[13] Truini A (2015). Trigeminal neuralgia. J Headache Pain.

[14] Olesen J (2005). The International Classification of Headache Disorders, 2nd edition: application to practice. Funct Neurol.

[15] Lambru G, Zakrzewska J, Matharu M (2021). Trigeminal neuralgia: a practical guide. Pract Neurol.

[16] Noguchi T, Shimamoto Y, Fukuda KI (2021). Clinical characteristics of trigeminal neuralgia in a dental hospital. J Dent Anesth Pain Med.

[17] Maarbjerg S, Gozalov A, Olesen J, Bendtsen L (2014). Concomitant persistent pain in classical trigeminal neuralgia--evidence for different subtypes. Headache.

[18] Rasmussen P (1990). Facial pain II. A prospective survey of 1052 patients with a view of: character of the attacks, onset, course, and character of pain. Acta Neurochir (Wien).

[19] Katusic S, Beard CM, Bergstralh E, Kurland LT (1990). Incidence and clinical features of trigeminal neuralgia, Rochester, Minnesota, 1945-1984. Ann Neurol.

[20] Ayele BA, Zewde YZ, Degefa MT, Mengesha AT (2021). Data set for demography, clinical characteristics, and risk factors of trigeminal neuralgia patients in Addis Ababa, Ethiopia. BMC Res Notes.

[21] Rehman A, Abbas I, Khan SA, Ahmed E, Fatima F, Anwar SA (2013). Spectrum of trigeminal neuralgia. J Ayub Med Coll Abbottabad.

[22] Mason WE, Kollros P, Jannetta PJ (1991). Trigeminal neuralgia and its treatment in a 13-month-old child: a review and case report. J Craniomandib Disord.

[23] Bender MT, Pradilla G, James C, Raza S, Lim M, Carson BS (2011). Surgical treatment of pediatric trigeminal neuralgia: case series and review of the literature. Childs Nerv Syst.

[24] Jainkittivong A, Aneksuk V, Langlais RP (2012). Trigeminal neuralgia: a retrospective study of 188 Thai cases. Gerodontology.

[25] Hall GC, Carroll D, Parry D, McQuay HJ (2006). Epidemiology and treatment of neuropathic pain: the UK primary care perspective. Pain.

[26] Ayele BA, Mengesha AT, Zewde YZ (2020). Clinical characteristics and associated factors of trigeminal neuralgia: experience from Addis Ababa, Ethiopia. BMC Oral Health.

[27] Araya EI, Claudino RF, Piovesan EJ, Chichorro JG (2020). Trigeminal neuralgia: basic and clinical aspects. Curr Neuropharmacol.

[28] Rozen TD (2004). Trigeminal neuralgia and glossopharyngeal neuralgia. Neurol Clin.

[29] Hooge JP, Redekop WK (1995). Trigeminal neuralgia in multiple sclerosis. Neurology.

[30] Zheng H, Shi YZ, Liang JT, Lu LL, Chen M (2023). Modifiable factors for migraine prophylaxis: a Mendelian randomization analysis. Front Pharmacol.

[31] Wei X, Zhou H, Zhang S, Hu X, Wei Z, Li Y (2023). A comprehensive two-sample Mendelian randomization analysis of trigeminal neuralgia and modifiable risk factors. Front Neurol.

[32] Cheng YH, Wu CH, Wang WT, Lu YY, Wu MK (2022). Trigeminal neuralgia is a dementia risk factor: a retrospective cohort study. Int J Environ Res Public Health.

[33] Xu Z, Zhang P, Long L, He H, Zhang J, Sun S (2016). Diabetes mellitus in classical trigeminal neuralgia: a predisposing factor for its development. Clin Neurol Neurosurg.

[34] Su YF, Wu CH, Wang WT, Lieu AS (2022). The risk of trigeminal neuralgia following osteoporosis. Medicina (Kaunas).

[35] Ali A, Bastianon Santiago R, Isidor J (2023). Debilitating trigeminal neuralgia secondary to idiopathic intracranial hypertension. Heliyon.

[36] Teruel A, Ram S, Kumar SK, Hariri S, Clark GT (2009). Prevalence of hypertension in patients with trigeminal neuralgia. J Headache Pain.

[37] Maarbjerg S, Gozalov A, Olesen J, Bendtsen L (2014). Trigeminal neuralgia--a prospective systematic study of clinical characteristics in 158 patients. Headache.

[38] Brisman R (2013). Constant face pain in typical trigeminal neuralgia and response to γ knife radiosurgery. Stereotact Funct Neurosurg.

[39] Obermann M, Yoon MS, Sensen K, Maschke M, Diener HC, Katsarava Z (2008). Efficacy of pregabalin in the treatment of trigeminal neuralgia. Cephalalgia.

[40] Bennetto L, Patel NK, Fuller G (2007). Trigeminal neuralgia and its management. BMJ.

[41] Tanrikulu L, Hastreiter P, Bassemir T, Bischoff B, Buchfelder M, Dörfler A, Naraghi R (2016). New clinical and morphologic aspects in trigeminal neuralgia. World Neurosurg.

[42] Sinay VJ, Bonamico LH, Dubrovsky A (2003). Subclinical sensory abnormalities in trigeminal neuralgia. Cephalalgia.

[43] Nurmikko TJ (1991). Altered cutaneous sensation in trigeminal neuralgia. Arch Neurol.

[44] Bowsher D, Miles JB, Haggett CE, Eldridge PR (1997). Trigeminal neuralgia: a quantitative sensory perception threshold study in patients who had not undergone previous invasive procedures. J Neurosurg.

[45] Younis S, Maarbjerg S, Reimer M, Wolfram F, Olesen J, Baron R, Bendtsen L (2016). Quantitative sensory testing in classical trigeminal neuralgia-a blinded study in patients with and without concomitant persistent pain. Pain.

[46] Zakrzewska JM, Jensen TS (2017). History of facial pain diagnosis. Cephalalgia.

[47] Di Stefano G, Maarbjerg S, Nurmikko T, Truini A, Cruccu G (2018). Triggering trigeminal neuralgia. Cephalalgia.

[48] Zakrzewska JM (2013). Differential diagnosis of facial pain and guidelines for management. Br J Anaesth.

[49] Zakrzewska JM, McMillan R (2011). Trigeminal neuralgia: the diagnosis and management of this excruciating and poorly understood facial pain. Postgrad Med J.

[50] Nurmikko TJ, Eldridge PR (2001). Trigeminal neuralgia--pathophysiology, diagnosis and current treatment. Br J Anaesth.

[51] Finsterer J (2024). SARS-CoV-2 vaccination- or infection-related trigeminal neuralgia/radiculitis. Intern Med.

[52] Schiffman E, Ohrbach R, Truelove E (2014). Diagnostic criteria for temporomandibular disorders (DC/TMD) for clinical and research applications: recommendations of the International RDC/TMD Consortium Network* and Orofacial Pain Special Interest Group†. J Oral Facial Pain Headache.

[53] List T, Jensen RH (2017). Temporomandibular disorders: Old ideas and new concepts. Cephalalgia.

[54] Boggero IA, Pickerill HM, King CD (2022). Fatigue in adults with chronic arthralgia/myalgia in the temporomandibular region: associations with poor sleep quality, depression, pain intensity, and future pain interference. J Oral Facial Pain Headache.

[55] Al-Khazali HM, Christensen RH, Lambru G, Dodick DW, Ashina H (2023). Hemicrania continua: an update. Curr Pain Headache Rep.

[56] Borges A, Casselman J (2010). Imaging the trigeminal nerve. Eur J Radiol.

[57] Ha SM, Kim SH, Yoo EH (2012). Patients with idiopathic trigeminal neuralgia have a sharper-than-normal trigeminal-pontine angle and trigeminal nerve atrophy. Acta Neurochir (Wien).

[58] Edvinsson L (2011). Tracing neural connections to pain pathways with relevance to primary headaches. Cephalalgia.

[59] Marinković S, Gibo H, Todorović V, Antić B, Kovacević D, Milisavljević M, Cetković M (2009). Ultrastructure and immunohistochemistry of the trigeminal peripheral myelinated axons in patients with neuralgia. Clin Neurol Neurosurg.

[60] Liu X, Ge X, Tang X (2023). Brain entropy changes in classical trigeminal neuralgia. Front Neurol.

[61] Alper J, Shrivastava RK, Balchandani P (2017). Is there a magnetic resonance imaging-discernible cause for trigeminal neuralgia? A structured review. World Neurosurg.

[62] Gospodarev V, Chakravarthy V, Harms C (2018). Computed tomography cisternography for evaluation of trigeminal neuralgia when magnetic resonance imaging is contraindicated: case report and review of the literature. World Neurosurg.

[63] Frisardi G, Chessa G, Sau G, Frisardi F (2010). Trigeminal electrophysiology: a 2 x 2 matrix model for differential diagnosis between temporomandibular disorders and orofacial pain. BMC Musculoskelet Disord.

[64] Lambru G, Matharu MS (2014). SUNCT, SUNA and trigeminal neuralgia: different disorders or variants of the same disorder?. Curr Opin Neurol.

[65] Schindler EA, Burish MJ (2022). Recent advances in the diagnosis and management of cluster headache. BMJ.

[66] Burish MJ, Rozen TD (2019). Trigeminal autonomic cephalalgias. Neurol Clin.

[67] Brandt RB, Haan J, Ferrari MD, Fronczek R (2020). [Cluster headache and other trigeminal autonomic cephalalgias]. Ned Tijdschr Geneeskd.

[68] Ramesh VG, Premkumar G (2009). An anatomical study of the neurovascular relationships at the trigeminal root entry zone. J Clin Neurosci.

[69] Chua KC, Kroetz DL (2017). Genetic advances uncover mechanisms of chemotherapy-induced peripheral neuropathy. Clin Pharmacol Ther.

[70] Murray D, Dilli E (2019). Primary stabbing headache. Curr Neurol Neurosci Rep.

[71] Schwedt TJ (2015). Thunderclap headache. Continuum (Minneap Minn).

[72] Hoffmann J, May A (2018). Diagnosis, pathophysiology, and management of cluster headache. Lancet Neurol.

[73] Cheng YF, Xirasagar S, Yang TH, Wu CS, Kao YW, Shia BC, Lin HC (2020). Increased risk of tinnitus following a trigeminal neuralgia diagnosis: a one-year follow-up study. J Headache Pain.

[74] Benoliel R, Zini A, Khan J, Almoznino G, Sharav Y, Haviv Y (2016). Trigeminal neuralgia (part II): factors affecting early pharmacotherapeutic outcome. Cephalalgia.

[75] Zakrzewska JM (2002). Diagnosis and differential diagnosis of trigeminal neuralgia. Clin J Pain.

[76] Di Stefano G, La Cesa S, Truini A, Cruccu G (2014). Natural history and outcome of 200 outpatients with classical trigeminal neuralgia treated with carbamazepine or oxcarbazepine in a tertiary centre for neuropathic pain. J Headache Pain.

[77] Heinskou TB, Maarbjerg S, Wolfram F, Rochat P, Brennum J, Olesen J, Bendtsen L (2019). Favourable prognosis of trigeminal neuralgia when enrolled in a multidisciplinary management program - a two-year prospective real-life study. J Headache Pain.

[78] Diener HC, Tassorelli C, Dodick DW (2023). Management of trigeminal autonomic cephalalgias including chronic cluster: a review. JAMA Neurol.

[79] Di Stefano G, Maarbjerg S, Truini A (2019). Trigeminal neuralgia secondary to multiple sclerosis: from the clinical picture to the treatment options. J Headache Pain.

[80] Truini A, Prosperini L, Calistri V (2016). A dual concurrent mechanism explains trigeminal neuralgia in patients with multiple sclerosis. Neurology.

[81] Nurmikko TJ, Gupta S, Maclver K (2010). Multiple sclerosis-related central pain disorders. Curr Pain Headache Rep.

[82] Katusic S, Williams DB, Beard CM, Bergstralh EJ, Kurland LT (1991). Epidemiology and clinical features of idiopathic trigeminal neuralgia and glossopharyngeal neuralgia: similarities and differences, Rochester, Minnesota, 1945-1984. Neuroepidemiology.

[83] Cruccu G, Gronseth G, Alksne J (2008). AAN-EFNS guidelines on trigeminal neuralgia management. Eur J Neurol.

[84] Broggi G, Ferroli P, Franzini A, Nazzi V, Farina L, La Mantia L, Milanese C (2004). Operative findings and outcomes of microvascular decompression for trigeminal neuralgia in 35 patients affected by multiple sclerosis. Neurosurgery.

[85] Truini A, Barbanti P, Pozzilli C, Cruccu G (2013). A mechanism-based classification of pain in multiple sclerosis. J Neurol.

[86] Athanasiou TC, Patel NK, Renowden SA, Coakham HB (2005). Some patients with multiple sclerosis have neurovascular compression causing their trigeminal neuralgia and can be treated effectively with MVD: report of five cases. Br J Neurosurg.

[87] Eldridge PR, Sinha AK, Javadpour M, Littlechild P, Varma TR (2003). Microvascular decompression for trigeminal neuralgia in patients with multiple sclerosis. Stereotact Funct Neurosurg.

[88] Kister I, Caminero AB, Monteith TS (2010). Migraine is comorbid with multiple sclerosis and associated with a more symptomatic MS course. J Headache Pain.

[89] Mrabet S, Wafa M, Giovannoni G (2022). Multiple sclerosis and migraine: links, management and implications. Mult Scler Relat Disord.

[90] Olesen J, Burstein R, Ashina M, Tfelt-Hansen P (2009). Origin of pain in migraine: evidence for peripheral sensitisation. Lancet Neurol.

[91] Lambru G, Stubberud A, Rantell K, Lagrata S, Tronvik E, Matharu MS (2021). Medical treatment of SUNCT and SUNA: a prospective open-label study including single-arm meta-analysis. J Neurol Neurosurg Psychiatry.

[92] VanderPluym J, Richer L (2015). Tic versus TAC: differentiating the neuralgias (trigeminal neuralgia) from the cephalalgias (SUNCT and SUNA). Curr Pain Headache Rep.

[93] Terrier LM, Hadjikhani N, Velut S (2021). The trigeminal system: the meningovascular complex-a review. J Anat.

[94] May A, Goadsby PJ (1999). The trigeminovascular system in humans: pathophysiologic implications for primary headache syndromes of the neural influences on the cerebral circulation. J Cereb Blood Flow Metab.

[95] Goadsby PJ (2001). Neuroimaging in headache. Microsc Res Tech.

[96] Terrier LM, Fontaine D (2021). Intracranial nociception. Rev Neurol (Paris).

[97] Penfield W, McNaughton F (1940). Dural headache and innervation of the dura mater. Arch NeurPsych.

[98] Borsook D, DaSilva AF, Ploghaus A, Becerra L (2003). Specific and somatotopic functional magnetic resonance imaging activation in the trigeminal ganglion by brush and noxious heat. J Neurosci.

[99] Dong B, Xu R, Lim M (2023). The pathophysiology of trigeminal neuralgia: a molecular review. J Neurosurg.

[100] Chen Q, Yi DI, Perez JN (2022). The molecular basis and pathophysiology of trigeminal neuralgia. Int J Mol Sci.

[101] Yuan R, Tong Z, Xiang G, Xie Y, Li K, Zhang L, Wang X (2023). The burden and trends of headache disorders among the population aged 15-39: a study from 1990 to 2019. J Headache Pain.

[102] Stovner Lj, Hagen K, Jensen R (2007). The global burden of headache: a documentation of headache prevalence and disability worldwide. Cephalalgia.

[103] Saylor D, Steiner TJ (2018). The global burden of headache. Semin Neurol.

